# Editorial Changes

**Published:** 1849

**Authors:** 


### ARTICLE IX.

### EDITORIAL CHANGES.

Dr. Drake the able pioneer of Medical Journalism in the west, has retired from the editorial charge of the Western Jour. of Med. and Surg. He has been a free, pleasing, and strong writer, and we part with him with regret, as loosing an editorial father.

Our promising young friend, and quandam class-mate, Dr. Colescott too has retired, leaving that paper exclusively in the hands of Prof. Yandall.

Dr. Huston of the Medical Examiner, so long and favourably known among the Editorial Corps, has retired from the editorial chair, and Drs. F. G. Smith, and D. H. Tucker, take his place.

We observe by the Buffalo Med. Jour., that a prospectus has been issued for pulishing the Western Medical Magazine at Cleveland O. Professor J. J. Delamater is to be the editor.

Professor Harrison has retired from the New Orleans Med. Jour.

